# Bridging access and impact: primary care parenting intervention reduces early behavior problems in both virtual and in-person delivery modes

**DOI:** 10.3389/fped.2026.1709280

**Published:** 2026-02-11

**Authors:** Fithi Andom, Joanne N. Wood, Li Jiang, Feng-Chang Lin, Samantha Schilling

**Affiliations:** 1 The Cecil G. Sheps Center for Health Services Research, University of North Carolina at Chapel Hill, Chapel Hill, NC, United States; 2 PolicyLab and Clinical Futures, Department of Pediatrics, Children’s Hospital of Philadelphia, Perelman School of Medicine at the University of Pennsylvania, Philadelphia, PA, United States; 3 Department of Biostatistics, Gillings School of Global Public Health, University of North Carolina at Chapel Hill, Chapel Hill, NC, United States; 4 Division of General Pediatrics and Adolescent Medicine, Department of Pediatrics, University of North Carolina at Chapel Hill, Chapel Hill, NC, United States

**Keywords:** child maltreatment prevention, early childhood behavior problems, parenting program, PriCARE, primary care, virtual intervention

## Abstract

**Background:**

Early childhood behavior problems are common and linked to adverse outcomes, including risk of maltreatment. Child-Adult Relationship Enhancement in primary care (PriCARE) is an evidence-based group parenting program delivered in pediatric primary care to reduce disruptive behaviors and strengthen caregiver-child relationships. In-person RCTs have demonstrated the efficacy of PriCARE, but barriers such as workforce shortages, transportation issues, and limited behavioral health infrastructure restrict access. Virtual delivery offers a potential solution, yet its effectiveness relative to in-person delivery is not well established.

**Objective:**

To evaluate the effectiveness of virtual PriCARE in improving child behavioral outcomes and compare these outcomes with those from prior in-person trials.

**Study design:**

A multi-center RCT of virtual PriCARE is underway with caregivers of children aged 18 months to 6 years. Child behavior was assessed using the Eyberg Child Behavior Inventory (ECBI) at baseline and at 6-8 month follow-up. An interim analysis was conducted to examine changes in ECBI scores from baseline to follow-up among virtual participants and to compare mean ECBI change trajectories between virtual delivery and prior in-person trials. Attendance patterns were compared using the Cochran-Armitage trend test. Effectiveness was evaluated using linear regression models with ANCOVA adjustment for baseline ECBI scores and caregiver/child demographics.

**Results:**

Subjects included 698 virtual PriCARE participants and 417 in-person PriCARE participants. Attendance was higher virtually, with 23.8% of participants completing all sessions, compared to 18.9% in-person (*p* < .001). Children in the virtual intervention group showed significant reductions in ECBI Intensity (−7.81 vs. 1.45, *p* < 0.001) and Problem scores (−3.80 vs. −1.91, *p* < .001) compared with usual care. The delivery mode×intervention interaction was not significant for either ECBI Intensity (*p* = 0.833) or Problem scores (*p* = 0.744), suggesting no evidence of differential effects by delivery mode.

**Conclusions:**

Virtual PriCARE was associated with significant improvements in early childhood behavior problems and higher completion rates, with no evidence of differential effects by delivery mode. These findings highlight the potential of virtual behavioral interventions in pediatric primary care to expand reach, reduce access barriers, and provide scalable prevention strategies to promote child well-being and prevent maltreatment.

**Trial registration:**

ClinicalTrials.gov, NCT05233150. Registered 10 February 2022 https://clinicaltrials.gov/study/NCT05233150.

## Introduction

Early childhood behavior problems are among the most common concerns reported in pediatric settings. Prevalence estimates suggest that 10%–23% of young children exhibit clinically significant disruptive behaviors such as aggression, defiance, and tantrums (1, 2). These behaviors are not only stressful for families but are also associated with long-term adverse outcomes, including academic difficulties, social challenges, and mental health problems in adolescence and adulthood (3, 4). Moreover, persistent behavior problems in early childhood are recognized as a key risk factor for child maltreatment, highlighting the importance of early identification and intervention (5, 6). Addressing these issues during the formative years is critical, as early support can alter developmental trajectories, reduce family stress, and prevent escalation of behavioral and safety risks (7–10).

Primary care provides a unique and strategic setting for addressing early behavior problems (11). Pediatric visits are frequent and routine during early childhood, offering regular contact with families and opportunities for early identification of behavioral concerns (12). They also provide an ideal opportunity to embed evidence-based parenting interventions, enabling providers to support caregivers in managing behavioral concerns early, before they escalate (13, 14). Such interventions not only improve child behavior outcomes but may also reduce caregiver stress and address other risk factors associated with child maltreatment (15, 16). Delivering these programs within primary care also enhances accessibility and engagement, particularly when barriers such as transportation, scheduling, or caregiver availability limit participation in traditional community-based programs (13).

One promising model for addressing early childhood behavior problems is Child-Adult Relationship Enhancement in Primary Care (PriCARE). PriCARE is a six-session evidence-based parenting intervention designed to equip caregivers with concrete strategies to manage disruptive behaviors, foster positive parent-child interactions, and strengthen attachment (17). PriCARE is grounded in a theory of change that links early disruptive behaviors to elevated parenting stress and increased risk of child maltreatment, with the parent-child relationship positioned as a key mechanism of intervention (16). Specifically, PriCARE targets caregiver use of positive reinforcement, consistent limit-setting, and emotion coaching to strengthen parent-child interactions, thereby reducing child behavior problems and preventing escalation into maltreatment. Prior PriCARE trials have consistently demonstrated improvements in child behavior and parenting practices (15, 17, 18).

Growing evidence also supports the effectiveness of parenting interventions delivered virtually. Recent meta-analyses show that parenting programs delivered through telehealth or digital formats produce child behavior improvements comparable to those delivered in person, particularly when programs include structured skills training and caregiver support. For example, a 2020 meta-analysis of 15 randomized trials found no significant differences in behavior outcomes between online and in-person parenting programs (10), and other systematic reviews similarly conclude that telehealth parent-training models yield equivalent reductions in child disruptive behaviors and improvements in parenting practices (19). These findings highlight the potential for virtual delivery to maintain intervention effectiveness while increasing accessibility for families facing structural barriers to in-person participation.

PriCARE was originally designed for delivery within pediatric primary care clinics through in-person group sessions. During the COVID-19 pandemic, PriCARE was adapted for virtual delivery to maintain access and engagement amid public health restrictions (20, 21) and is currently being evaluated in a large, randomized control trial (RCT) (22). Given the shift in delivery mode from in-person to virtual, a planned mid-study analysis was incorporated to assess whether virtual delivery affects intervention effectiveness, and to determine whether delivery should return to in-person for the second half of the trial. Understanding the comparative impact of in-person vs. virtual delivery modes is particularly important in the current landscape of expanding telehealth services, as well as the goal of increasing access for families facing barriers to in-person participation.

In this study, we report the findings from the mid-study analysis of the virtual PriCARE trial (22). Focusing on child behavior outcomes, this analysis addresses two research questions: (a) Is the current virtual PriCARE trial effective in improving child behavior outcomes? and (b) Compared with previous in-person trials (15, 17, 18), does virtual delivery of PriCARE produce comparable or greater treatment effects? By examining these questions, this study assesses both the continued effectiveness of PriCARE in a contemporary sample and the potential of telehealth adaptations to maintain or enhance the intervention impact. By comparing mid-study child behavior outcomes to historical in-person results, this study offers important insights into whether PriCARE can maintain its effectiveness while increasing accessibility.

## Materials and methods

### Study design and population

This study presents a mid-study analysis of an ongoing randomized controlled trial (RCT) evaluating the virtual delivery of PriCARE (22). The trial employs a parallel-group design, in which caregiver-child dyads are randomly assigned 1:1 to either PriCARE plus usual care or usual care alone, defined as routine care from their primary care provider (hereafter also referred to as the control group). The current analysis compares changes in child behavior outcomes for the first 698 dyads enrolled in virtual PriCARE with outcomes from 417 families who participated in three prior in-person RCTs. A CONSORT diagram for the virtual trial is provided in Supplementary Figure D1. CONSORT diagrams for the in-person trials are available in the original publications (15, 17, 18).

Eligibility requirements and randomization procedures were similar but not identical across all four trials (see Table 1). Notable differences in the current virtual trial include: child age eligibility (18 months to 6 years, compared to 2-6 years in the in-person trials), inclusion of families with Medicaid or no insurance (vs. any insurance type in previous trials), and the enrollment of both Spanish- and English-speaking caregivers (compared to English-only in prior studies). The 2017 and 2021 in-person trials required caregivers to report a perceived child behavior problem for eligibility, whereas the 2020 and current virtual trials did not. In addition, the current trial was conducted as a multicenter study across both the Children's Hospital of Philadelphia (CHOP) and the University of North Carolina (UNC), recruiting from numerous practices within each system. In contrast, prior studies were single-site and recruited from only a few practices.

**Table 1:** Inclusion and exclusion criteria, randomization methods, follow-up duration, attrition, and sample sizes across PriCARE randomized controlled trials conducted in pediatric primary care.

Study	Inclusion criteria	Exclusion criteria	Randomization	Time to follow-up (SD)	*N* (%) lost to follow-up	*N* Total randomized (intervention/control)
Schilling et al., (17)	Caregiver of child aged 2–6 years with behavior concerns; Primary care at participating site; English fluency	Child with cognitive delays resulting in functioning below a 2-year-old level; engaged in therapy or prescribed medication for behavioral health problem	Randomized 2:1 to PriCARE or usual care	3.8 (0.56) months	0 (0%)	120 (80/40)
Schilling et al., (18)	Caregiver of child aged 2–6 years; Primary care at participating site; English fluency; Willingness for peer mentor involvement	Child with cognitive delays resulting in functioning below a 2-year-old level; child receiving medical treatment for a behavior disorder other than attention deficit hyperactivity disorder	Randomized 1:1:1 to PriCARE, mentored PriCARE or control	3.19 (1.66) months	3 (2%)	150 (100/50)
Wood et al., (15)	Caregiver of child aged 2–6 years with behavior concerns; Primary care at participating site; English fluency	Same as 2020 trial	Randomized 2:1 to PriCARE or usual care	4.8 (1.4) months	18 (10.3%)	174 (119/55)
Schilling et al., (22) (Current Virtual Trial)	Caregiver of child aged 18 months-6 years; Primary care at participating site; English or Spanish fluency; Internet-enabled device access; Medicaid insurance or no insurance	Child with no receptive language (cannot follow a command)	Randomized 1:1 to virtual PriCARE or usual care	6.52 (0.65) months	52 (6.8%)	698 (344/354)

PriCARE, primary care-based child-adult relationship enhancement. Follow-up time is reported in months (mean ± SD). Attrition reflects participants lost to follow-up by final assessment. Usual care refers to standard pediatric primary care without PriCARE intervention.

Randomization procedures varied across studies, which influenced the proportion of participants assigned to the intervention and control groups. The 2017 and 2021 trials employed a 2:1 randomization protocol (PriCARE vs. control). The 2020 trial included three arms -PriCARE, mentored PriCARE, and control, with participants randomized equally in a 1:1:1 ratio. The current 2023 virtual trial employs stratified block randomization by clinical site and language, with a 1:1 allocation ratio (Table 1). These variations in randomization methods explain differences in group sizes across trials and are accounted for in the comparative analysis. Across all trials, caregivers were recruited during well-child visits at pediatric primary care clinics using an embedded alert system in the electronic medical record, in addition to other recruitment methods such as flyers posted in clinical settings and emails to potential caregiver subjects.

To determine whether virtual delivery yields outcomes comparable to traditional in-person implementation, we compare child behavior results from the current trial with those reported in the three previous PriCARE trials (15, 17, 18). Although multiple child and caregiver outcomes were assessed across these studies, the Eyberg Child Behavior Inventory (ECBI) was the only measure used in all four trials. Accordingly, the ECBI is the focus of this comparative analysis. Table 2 provides an overview of outcome measures used across the four trials.

**Table 2:** Overview of outcome measures used across PriCARE randomized controlled trials.

Outcome domain	Schilling et al., (17)	Schilling et al., (18)	Wood et al., (15)	Schilling et al., (10)
Child behavior	Eyberg child behavior inventory (ECBI)	ECBI	ECBI	ECBI
Discipline	Adult adolescent parenting inventory 2 (AAPI2)	Parenting scale (PS)	AAPI2	PS conflict tactics scale- parent-child version
Parent stress	Not measured	Not measured	Parenting stress index- short form (PSI-SF)	PSI-SF
Child maltreatment	Not measured	Not measured	Not measured	Child protective services investigations brief child abuse potential inventory

This table summarizes the primary outcomes measures used in each PriCARE trial. ECBI, eyberg child behavior inventory; AAP12, adult-adolescent parenting intventory-2; PS, parenting scale; PSF-SF, parenting stress index—short form; CTS-PC, conflict tactics scale—parent-child version; BCAP, brief child abuse potential inventory. Cells indicate which instrument was administered in each study. Blank cells indicate that the outcome was not measured.

### PriCARE intervention

PriCARE is a trauma-informed, evidence-based group parenting intervention adapted from the CARE model (18). It consists of six structured sessions that teach caregivers practical strategies to manage disruptive behaviors, enhance positive interactions, and build stronger attachment with their child. The core skills taught are Praise (specific positive reinforcement), Paraphrase (active listening and validation), and Point-out (descriptive commenting to increase engagement and reduce conflict). These skills are designed to disrupt coercive parent-child cycles that contribute to child behavior problems and parenting stress. Sessions incorporate brief didactics, interactive skill practice, and home assignments to reinforce learning.

Originally, PriCARE was designed for recruitment and delivery within pediatric primary care clinics. Pediatricians introduced the intervention and made referrals during well-child visits. The intervention was delivered in small groups of 4-10 caregivers over six in-person sessions led by two trained therapists. These 1.5-hour sessions typically took place in a clinic conference room and were supported with childcare, meals, and transportation reimbursement to reduce barriers to participation. For the current trial, PriCARE is delivered virtually via secure video conferencing. Virtual delivery retained the full structure and content of the original in-person curriculum but incorporated several adaptations to optimize engagement and accessibility. These adaptations included mailed physical handouts and activity materials, digital homework reminders, technical support during sessions, and flexible make-up options via asynchronous video to augment missed live sessions. Make-up videos are unique to the virtual delivery mode and were not offered in the prior in-person trials. These modifications were developed and tested in a previous feasibility study, which found the virtual delivery mode to be acceptable and usable for most families (20). The full virtual intervention protocol is described elsewhere (22).

### Measures

The primary outcome, child behavior, was assessed using the ECBI, a widely used and validated parent-report measure of disruptive behavior in children aged 2–16 years (23). The ECBI includes two subscales: the Intensity Scale, which rates the frequency of 36 problem behaviors on a 7-point Likert scale (1 = never to 7 = always), and the Problem Scale, which assesses whether each behavior is perceived as a problem by the parent (yes/no). A total Intensity score above 130 is generally considered clinically significant, indicating a high frequency of child behavioral problems that may need intervention. For the Problem Scale, scores range from 0 to 36, with a cutoff of 15 or higher commonly used as a threshold for clinical concern, suggesting that caregivers identify multiple behaviors as problematic (24). The ECBI has shown strong psychometric properties, with Cronbach's alpha values ranging from 0.93 to 0.98 across different populations (25, 26). The tool's construct validity is also supported by strong correlations with externalizing behavior subscales of established instruments such as the Child Behavior Checklist (27, 28). In all four studies, the ECBI was administered at two time points:- baseline (time 1) and follow-up (time 2), and change scores were calculated. The follow-up window varied across studies: in the 2017 trial, follow-up occurred at 3.8 ± 0.56 months; in the 2020 trial, follow-up occurred at 3.19 ± 1.66 months; in the 2021 trial follow-up occurred at 4.8 ± 1.4 months; and in the virtual trial, follow-up occurred at 6.52 ± 0.65 months.

Additional demographic and family characteristics, including child age, sex, race/ethnicity, and caregiver sex, were included to adjust for potential confounders. Caregiver session attendance was also recorded to assess program engagement and for a more holistic understanding of the intervention's impact and accessibility across delivery modes.

### Statistical analysis

Attendance across study groups and delivery modes was analyzed using the Cochran-Armitage trend test, which evaluates trends in categorical outcomes. To assess the effectiveness of the virtual PriCARE trial, changes in ECBI Intensity and Problem scores from baseline to follow-up were analyzed using two-sample t-tests and analysis of covariance (ANCOVA). The dependent variables were the change in ECBI scores, with baseline ECBI scores included as covariates. Additional covariates in the adjusted model included child age, sex, race/ethnicity, caregiver sex and study site. Both unadjusted (crude) and adjusted models are presented, with mean changes in scores, 95% confidence intervals, and *p*-values reported.

To examine whether intervention effects differed by delivery mode, we used linear regression models that included the baseline ECBI score, child demographics, caregiver sex, delivery mode (virtual vs. in-person), study arm, and the delivery mode × study arm interaction term. This interaction term tested whether the magnitude of the intervention effect varied as a function of delivery mode. A non-significant interaction was interpreted as indicating no evidence of differential intervention effects between virtual and in-person delivery. ECBI Intensity and Problem scale scores were calculated according to standard ECBI scoring guidelines. When fewer than four items were missing on a scale, missing Intensity items were assigned a value of 1 (“never”), and missing Problem items were assigned a value of 0 (“not a problem”). Scales with four or more missing items were considered invalid, and participants with invalid scores were excluded from the analyses. Statistical significance was set at *p*-value < 0.05, and effect sizes with 95% confidence intervals were reported for all primary outcomes. Additional analytic details, including independent-samples *t*-tests, linear regression models, and the CONSORT flow diagram describing participant recruitment and retention for the ongoing virtual study, are provided in the Supplementary Materials. All analyses were conducted using R 4.3. (Vienna, Austria).

### Ethics approval statement

The study was approved by the University of North Carolina Institutional Review Board (Protocol No. 20-2605).

## Results

### Demographic characteristics of participants

A total of 1,115 caregiver-child dyads were included in the analysis, with 698 in the virtual PriCARE trial and 417 from the three in-person PriCARE trials (Table 3). Several demographic and baseline characteristics differed significantly between the virtual and in-person groups. A higher proportion of participants in the virtual trial were assigned to the control group (50.7%) compared to the in-person trials (32.9%), with a statistically significant difference (*p* < .001). The virtual participants also had a greater percentage of Hispanic children (31.7% vs. 13.3%) and a lower percentage of Black children (56.9% vs. 64.3%) (*p* < .001 for both ethnicity and race). The parent sex distribution approached significance, with a slightly higher proportion of female caregivers in the virtual group (97.9% vs. 95.7%, *p* = .06). Child age differed significantly between groups (*p* < 0.001), as the in-person studies included only children 2 years and older.

**Table 3:** Baseline demographic and clinical characteristics of caregiver-child dyads in in-person and virtual PriCARE randomized controlled trials.

Variable	In-Person (*N* = 417)	Virtual (*N* = 698)	*p*
Randomization group			<.001
Control	137 (32.9%)	354 (50.7%)	
Intervention	280 (67.1%)	344 (49.3%)	
Child sex			.84
Female	183 (43.9%)	312 (44.7%)	
Male	234 (56.1%)	386 (55.3%)	
Parent sex			.06
Female	399 (95.7%)	683 (97.9%)	
Male	18 (4.3%)	15 (2.1%)	
Child ethnicity			<.001
Hispanic	54 (12.9%)	221 (31.7%)	
Non-Hispanic	363 (87.1%)	477 (68.3%)	
Child race			<.001
Black	268 (64.3%)	397 (56.9%)	
Other	40 (9.6%)	138 (19.8%)	
White	109 (26.1%)	163 (23.4%)	
Child age (years)			<.001
*M* (SD)	3.68 (1.35)	3.30 (1.41)	
Median [range]	4.00 [2.00, 6.00]	3.00 [1.00, 6.00]	
ECBI Intensity baseline			<.001
*M* (SD)	132 (41.4)	114 (37.9)	
Median [range]	132 [39.0, 229]	114 [39.0, 242]	
ECBI Problem baseline			<.001
*M* (SD)	16.4 (8.75)	12.8 (8.47)	
Median [range]	17.0 [0, 36.0]	12.0 [0, 32.0]	

ECBI, eyberg child behavior inventory. Baseline measures were obtained prior to randomization. *P* values reflect comparisons between in-person and virtual trial samples.

### Baseline child behavior

Baseline scores on the ECBI differed significantly between cohorts. Children in the virtual trial had lower mean scores on both the Intensity and Problem scales at baseline (all *p* < .001), suggesting lower levels of disruptive behaviors were reported in this trial compared to the in-person trials (see Table 3).

### Attendance differences between delivery modes

Attendance differed significantly between in-person and virtual PriCARE participants (*p* *<* .001; Table 4). A higher proportion of caregivers in the virtual delivery mode attended all six sessions compared to the in-person mode (23.8% vs. 18.9%). At the same time, non-attendance was higher in the in-person mode (23.2% vs. 14.5%). When makeup videos were included, overall attendance differed significantly between delivery modes (*p* < 0.001), with 52.9% of virtual participants completing all six sessions, and non-attendance decreased to 0.3%. These findings highlight higher overall completion and lower attrition in the virtual delivery mode.

**Table 4:** Session attendance among intervention participants by delivery mode (in-person vs. virtual PriCARE).

Attendance	In-person (*N* = 280)	Virtual (*N* = 344)	*p* value	In-person (*N* = 280)	Virtual (*N* = 344)	*p* value
	Attendance only		<.001	Attendance + makeup videos		<.001
0	65 (23.2%)	50 (14.5%)		65 (23.2%)	1 (0.3%)	
1	37 (13.2%)	47 (13.7%)		37 (13.2%)	40 (11.6%)	
2	24 (8.6%)	44 (12.8%)		24 (8.6%)	44 (12.8%)	
3	26 (9.3%)	19 (5.5%)		26 (9.3%)	37 (10.8%)	
4	22 (7.9%)	37 (10.8%)		22 (7.9%)	23 (6.7%)	
5	53 (18.9%)	65 (18.9%)		53 (18.9%)	17 (4.9%)	
6	53 (18.9%)	82 (23.8%)		53 (18.9%)	182 (52.9%)	
Total	280 (100%)	344 (100%)		280 (100%)	344 (100%)	

Values represent frequencies with percentages in parentheses. Attendance reflects the number of PriCARE sessions attended (maximum = 6). “Attendance only” includes live session participation only, whereas “Attendance + makeup videos” includes completion of recorded makeup sessions. *P* values reflect comparisons between in-person and virtual intervention groups.

### Child behavior outcomes—current virtual study

In the current virtual PriCARE trial, children in the intervention group showed significantly greater improvements in behavior outcomes compared to those in the control group (Table 5). For the ECBI Intensity scale, the intervention group showed a mean change of −7.81 points [95% CI (−11.31, −4.30)], while the usual care group showed a mean change of 1.45 points [95% CI (−1.50, 4.39)]. The between-group difference in mean change was statistically significant in both crude (*p* < 0.001) and adjusted models (*p* < 0.001). On the ECBI Problem scale, the intervention group improved by −3.80 points [95% CI (−4.65, −2.96)] compared to −1.91 points [95% CI (−2.67, −1.16)] in the control group (*p* < .001, adjusted). Effect sizes were small to medium (Cohen's d = 0.30 for Intensity; Cohen's d = 0.26 for Problem), indicating modest improvements in child behavior among families receiving the intervention.

**Table 5:** Changes in eyberg child behavior inventory (ECBI) intensity and problem scores Among intervention and control participants in the in theVirtual PriCARE randomized controlled trial.

Outcome measure	Control (*N* = 354)	Intervention (*N* = 344)	Crude *p* value	Adjusted *p* value	Cohen's *d*
ECBI Intensity Change	1.45 [−1.50, 4.39]	−7.81 [−11.31, −4.30]	<.001	<.001	0.30
ECBI Problem Change	−1.91 [−2.67, −1.16]	−3.80 [−4.65, −2.96]	<.001	<.001	0.26

Values represent mean change scores with 95% confidence intervals in brackets. Negative values reflect reductions in child behavior problems. ECBI, Eyberg Child Behavior Inventory. Adjusted *p* values are from controlling for baseline ECBI score and prespecified covariates.

### Child behavior outcomes—across in-person and virtual delivery mode

When comparing virtual and in-person trials, both delivery modes of the PriCARE intervention resulted in significantly greater reductions in child behavior problems than their respective usual care groups (Table 6). For in-person delivery, the ECBI Intensity showed a mean change of −15.98 [95% CI: −19.67, −12.29] and the ECBI Problem showed a mean change of −4.08 [95% CI: −4.95, −3.20] in the intervention arm. For virtual delivery, the ECBI Intensity showed a mean change of −7.81 [95% CI: −11.31, −4.30] and the ECBI Problem showed a mean change of −3.84 [95% CI: −4.65, −3.03] for the intervention arm. Usual care groups in both delivery modes showed minimal changes. These values are presented for descriptive comparison only; no inferential statistical tests were conducted across historical cohorts, and effect sizes are not reported.

**Table 6:** Estimated changes in eyberg child behavior inventory (ECBI) intensity and problem scores by treatment condition and delivery mode in in-person and virtual PriCARE randomized controlled trials.

Outcome	Control in-person (*n* = 137)	Control virtual (*n* = 354)	Intervention in-person (*n* = 280)	Intervention virtual (*n* = 344)
ECBI intensity change	−3.75 (−8.56, 1.06)	1.45 (−1.50, 4.39)	−15.98 (−19.67, −12.29)	−7.81 (−11.31, −4.30)
ECBI problem change	−0.85 (−1.98, 0.26)	−1.91 (−2.67, −1.16)	−4.08 (−4.95, −3.20)	−3.84 (−4.65, −3.03)

Values are estimated mean changes from baseline (time 1) to follow-up (time 2), with 95% confidence intervals in parentheses. Table presents descriptive statistics only; inferential comparisons were not conducted, and effect sizes are therefore not reported.

**Figure 1:**
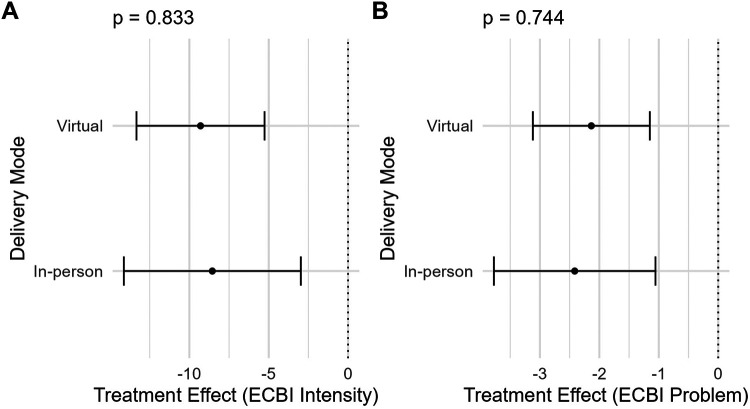
Comparison of virtual and in-person PriCARE intervention effects on child behavior outcomes. ECBI, Eyberg Child Behavior Inventory. Forest plots comparing estimated intervention effects for virtual and in-person PriCARE delivery modes on **(A)** ECBI Intensity scores and **(B)** ECBI Problem scores. Points represent estimated treatment effects, and horizontal lines indicate 95% confidence intervals. Negative values indicate greater reductions in child behavior problems. *P* values reflect tests of differences in treatment effects between delivery modes.

Linear regression models of change in ECBI scores, adjusting for baseline ECBI scores, child demographics, and caregiver sex and race/ethnicity found that the delivery mode × study arm interaction was not significant for either ECBI Intensity (*p* = 0.833) or ECBI Problem (*p* = 0.744) scales, indicating no evidence of differential treatment effects by delivery mode (Figure 1).

## Discussion

This mid-study analysis provides evidence that PriCARE remains effective in reducing early childhood behavior problems when delivered virtually. Children whose caregivers participated in the virtual intervention showed significantly greater improvements on the ECBI Intensity and Problem scales compared to the usual care group, even after adjusting for baseline scores and demographic variables. Effect sizes were small to medium (d = 0.26–0.30), indicating modest but meaningful improvements that are consistent with prior evaluations of parenting interventions. These findings align with prior in-person PriCARE trials, which have consistently documented reductions in disruptive behavior and improvements in parent-child relationships across diverse settings (15, 17–29). More broadly, our results are consistent with meta-analytic findings showing that evidence-based parenting programs reliably reduce child behavior problems across delivery models (10, 19). Although relatively few meta-analyses have focused explicitly on pediatric primary care delivered parenting programs, effect sizes from these clinic-based interventions (e.g., Standardized Mean Difference ≈ 0.29) (30) fall within the small-to-moderate range reported in broader parent training meta-analysis (e.g., Cohen's d ≈ 0.42) (31). These findings suggest that primary care delivery can achieve outcomes similar to community or home-based models, supporting the scaling of PriCARE in clinic settings while continuing to refine engagement strategies for underserved caregivers.

Notably, the study-arm x delivery mode interaction was not significant, indicating no evidence of differential treatment effects between virtual and in-person delivery. Although the virtual trial had a longer follow-up window compared to the in-person studies, the observed behavioral improvements were generally consistent across trials. Nonetheless, longer follow-up could contribute to modest decreases in effects over time, which should be considered when interpreting comparative results. With fewer instances of non-attendance, our findings align with the growing body of literature suggesting that telehealth adaptations of parenting programs can yield outcomes similar to those of in-person models when caregiver engagement and intervention fidelity are maintained (32). These findings also suggest that telehealth delivery can increase accessibility and sustain caregiver involvement, particularly among families facing barriers to in-person participation (33, 34).

There were notable systematic differences between the in-person and virtual samples that are important for interpreting comparative outcomes. The virtual cohort included a substantially higher proportion of Hispanic families and greater racial diversity, whereas the in-person sample had a larger proportion of Black participants. Children in the virtual group were also younger on average, and both ECBI Intensity and Problem baseline scores were significantly lower compared to the in-person sample. These demographic and clinical differences may represent meaningful contextual variations that influence responsiveness to PriCARE. For example, younger age and lower baseline behavioral severity, both characteristics of the virtual cohort, are associated with smaller absolute behavioral change in parent-training interventions (35–37). This pattern aligns with our findings, in which the virtual intervention group showed improvements in ECBI Intensity but with a smaller magnitude (−7.45) than the in-person intervention group (−15.69). Similarly, sample differences in racial/ethnic composition may point to cultural or structural moderators that shaped which families accessed in-person vs. virtual services. Although our analyses adjusted for several covariates, these systematic differences limit causal attribution to differences in outcomes solely to delivery format. Future studies should test whether age, baseline severity, and sociocultural context moderate PriCARE's effectiveness across delivery modes.

From a theoretical perspective, these findings are consistent with PriCARE's focus on reducing coercive parent-child interaction patterns and strengthening caregiver-child attachment. By teaching caregivers structured strategies for improving communication, managing behavior, and enhancing positive interactions, PriCARE targets well-established mechanisms of behavior change that are directly linked to reductions in child behavior problems (16). Engagement gains in the virtual PriCARE may have further reinforced these mechanisms by ensuring that more families received the full dosage of the intervention. In this way, improved attendance and reduced attrition may help explain how the program maintained effectiveness in the virtual mode despite the potential challenges of remote delivery.

The broader implications of these findings are noteworthy. First, the feasibility and effectiveness of virtual PriCARE highlights the potential of telehealth as a scalable model for addressing child behavior problems in early childhood. Workforce shortages, long waitlists, and geographic inequities often delay access to behavioral health services for young children. By delivering evidence-based interventions remotely, health systems can expand reach without overburdening the limited pool of trained clinicians (32–34, 38). These findings are consistent with emerging evidence that digital adaptations of behavioral interventions, when accompanied by active caregiver engagement, can effectively reduce child emotional and behavioral difficulties (39–41). The present study contributes to this literature by demonstrating that a group-based, evidence-informed parenting program can be delivered virtually while retaining therapeutic benefits and even enhancing caregiver engagement.

The adaptability of PriCARE also complements the expanding CARE literature, which documents the translational utility of CARE-based approaches across various settings and populations. For example, Swails et al. (42) demonstrated the feasibility of adapting CARE workshops for videoconferencing during the COVID-19 pandemic (TeleCARE), with strong caregiver engagement and promising preliminary outcomes. Loomis et al. (43) found that CARE training for early childhood providers enhanced trauma-informed attitudes and collaboration, suggesting that strengthening the relational skills of caregivers and providers can indirectly contribute to child maltreatment prevention. Similarly, Kiefer et al (44). documented positive perceptions of CARE training among educational, behavioral health, and allied health professionals, highlighting the translational potential of CARE-informed interventions across service systems and delivery modalities. The current findings extend this evidence base by showing that PriCARE, as a CARE adaptation embedded in primary care, retains its effectiveness when delivered virtually, further strengthening the case for its scalability.

Beyond immediate behavioral outcomes, the implications for child maltreatment prevention and family resilience are critical. Early behavior problems are well-established predictors of adverse developmental trajectories and maltreatment risk, particularly in families experiencing stress, caregiver mental health challenges, or limited access to support services (5, 6). By strengthening parent–child interactions, improving caregiver skills, and reducing coercive dynamics, PriCARE addresses proximal risk factors that often precede maltreatment. Delivering the program virtually increases its preventive potential by reaching families who might otherwise be excluded from in-person services due to logistical, financial, or geographic barriers. In this way, PriCARE demonstrates how early behavioral health interventions can function as upstream child maltreatment prevention strategies, advancing a more proactive and equitable approach.

These findings also carry broader policy and system-level implications. Embedding scalable, evidence-based interventions like PriCARE into routine service systems, including primary care, early education, and community service systems, can maximize their public health impact. Technology-facilitated delivery models reduce reliance on highly specialized clinicians, making it possible for interventions to be disseminated more widely across communities with limited behavioral health infrastructure. They also facilitate a more equitable distribution of preventive support by mitigating structural barriers to access, such as transportation challenges or the stigma associated with attending behavioral health services in clinical settings (45). Integrated within public health and child welfare frameworks, interventions like PriCARE can promote early detection, timely intervention, strengthen connections to community resources, and reduce the downstream risk of behavioral problems and maltreatment, thereby reinforcing child and family well-being within existing systems of care (46–48).

Primary care represents a strategic setting for delivering interventions like PriCARE. Pediatricians and family physicians maintain longitudinal relationships with families through well-child visits, offering a trusted and familiar platform for early identification of behavioral concerns and timely intervention. Integrating PriCARE into primary care settings leverages these routine touchpoints to reduce stigma, normalize behavioral health support, and promote early intervention (13–15). Moreover, primary care based delivery aligns with calls to integrate behavioral and physical health care to promote whole-child wellbeing (49). Virtual delivery extends these advantages by reducing geographic constraints, expanding reach into underserved areas, and offering flexibility that aligns with caregivers’ schedules. In this way, virtual PriCARE advances a model of preventive care that is accessible, family-centered, and integrated within familiar healthcare systems.

Several limitations should be considered when interpreting these findings. As a mid-study analysis of the ongoing large-scale RCT, our findings are preliminary and should be interpreted with caution. First, the present study relies on the ECBI as the sole outcome measure. Although widely used and validated, the ECBI is a parent-reported measure and may be subject to reporting bias. Second, the virtual cohort was compared with historical in-person trials rather than concurrently randomized controls, introducing the possibility of unmeasured confounding. Because virtual and in-person delivery were evaluated in separate cohorts, delivery mode was confounded with study and time, constraining direct causal comparisons across modes despite the absence of statistically significant interaction effects. Third, the study population may not fully represent the diversity of all primary care settings, which could potentially limit the generalizability of the findings.

Future analyses from the ongoing PriCARE trial will address several of these limitations. Specifically, the completed trial will assess validated measures of harsh and neglectful parenting, child abuse potential, and child welfare investigations, providing more direct evidence of PriCARE's impact on maltreatment prevention. Additionally, investigating mechanisms of change, such as enhancements in caregiver self-efficacy, reductions in parenting stress, and strengthened parent–child relationship, will inform refinements to the intervention and strategies for broader implementation. Future work will also evaluate cost-effectiveness, long-term behavioral outcomes, and sustainability of virtual delivery, which are essential for informing large-scale adoption. Evaluating these factors will be critical not only for translating PriCARE into routine clinical practice but also for shaping public health policy and guiding scalable approaches to early childhood behavioral health and maltreatment prevention.

## Conclusion

In summary, this study provides mid-trial evidence that PriCARE is effective in reducing early childhood behavioral problems when delivered virtually, with outcomes consistent with those observed in prior in-person trials. Virtual delivery not only maintains these positive outcomes but also improves accessibility by reducing attendance barriers and expanding reach. By addressing workforce shortages and geographic disparities, technology-facilitated interventions like PriCARE offer a scalable solution to improve early childhood behavioral health and proactively reduce maltreatment risk. Embedding such programs within primary care and other trusted systems provides a pathway to timely intervention. It promotes more equitable models of child and family wellbeing, especially for families who might otherwise be underserved. Collectively, these findings underscore the potential of virtual, evidence-based interventions to transform the landscape of early childhood behavioral health and inform policies aimed at building more responsive, preventive, and equitable systems of care.

## Data Availability

The data analyzed in this study are part of an ongoing randomized controlled trial funded by the Eunice Kennedy Shriver National Institute of Child Health and Human Development (NICHD) under NIH grant number (1R01HD103902-01). Due to the ongoing nature of the trial, the dataset is not publicly available at this time. Data sharing will be considered upon completion of the trial and publication of primary outcomes, in accordance with NIH data sharing policies. Requests for access prior to study completion may be directed to the corresponding author; however, availability will be restricted until the study is finalized. Requests to access the datasets should be directed to samantha_schilling@med.unc.edu.
