# Supplementary material for: Bridging access and impact: primary care parenting intervention reduces early behavior problems in both virtual and in-person delivery modes
Source: Front Pediatr. 2026 Feb 11;14:1709280. doi: 10.3389/fped.2026.1709280 (PMC12932534; doi:10.3389/fped.2026.1709280)
Supplement: Supplementary file 1 [file Datasheet1.pdf]

Appendix A

Raw Eyberg Child Behavior Inventory (ECBI) Scores by Intervention Delivery Mode and Study Condition

Table A1

Raw Baseline and Follow-up ECBI Intensity scores by Delivery Mode and Treatment Condition

Timepoint	Virtual Delivery		In-Person Delivery	
	Intervention	Control	Intervention	Control
n	344	354	280	137
Baseline Mean (SD)	113.67 (38.22)	113.88 (37.64)	135.60 (40.83)	126.08 (41.95)
Follow-up Mean (SD)	105.87 (34.49)	115.32 (35.76)	119.62 (38.73)	122.33 (41.94)

Note. Values represent mean scores with standard deviations in parenthesis. ECBI = Eyberg Child Behavior Inventory. Scores are reported at baseline (prior to randomization) and at follow-up.

Table A2

Raw Baseline and Follow-up ECBI Problem scores by Delivery Mode and Treatment Condition

Timepoint	Virtual Delivery		In-Person Delivery	
	Intervention	Control	Intervention	Control
n	344	354	280	137
Baseline Mean (SD)	12.50 (8.54)	13.01 (8.41)	17.09 (8.80)	15.14 (8.52)
Follow-up Mean (SD)	8.66 (7.97)	11.09 (8.62)	13.01 (8.80)	14.29 (9.29)

Note. Values represent mean scores with standard deviations in parenthesis. ECBI = Eyberg Child Behavior Inventory. Score are reported at baseline (prior to randomization) and at follow-up

Appendix B

Adjusted Linear Regression Models Examining Change in Eyberg Child Behavior Inventory (ECBI) Scores in the Virtual PriCARE Randomized Controlled Trial

Table B1

Adjusted Linear Regression Model Predicting Change in ECBI Intensity Scores in the Virtual PriCARE Trial

Predictor	B	SE	t	p	95% CI
(Intercept)	45.854	5.108	8.976	<0.001	[35.824, 55.884]
Baseline ECBI Intensity Score	-0.404	0.028	-14.315	<0.001	[-0.459, -0.348]
Intervention	-9.295	2.026	-4.587	<0.001	[-13.274, -5.316]
Child sex (Male)	3.335	2.045	1.631	0.103	[-0.681, 7.351]
Caregiver sex (Male)	4.815	7.002	0.688	0.492	[-8.933, 18.563]
Child ethnicity (Non-Hispanic)	3.693	2.717	1.359	0.175	[-1.642, 9.027]
Child race (Other)	-0.327	3.307	-0.099	0.921	[-6.819, 6.166]
Child race (White)	-1.915	2.619	-0.731	0.465	[-7.058, 3.228]
Child age	-0.862	0.729	-1.183	0.237	[-2.293, 0.569]
Study Site (UNC)	0.674	2.197	0.307	0.759	[-3.639, 4.988]

Note. B = unstandardized regression coefficients representing the estimated mean change in ECBI Intensity score associated with each predictor. Negative coefficients indicate greater reductions in child behavior problems. Model is adjusted for baseline ECBI Intensity score, child age, child sex, caregiver sex, child race, child ethnicity, and study site (UNC vs. CHOP). ECBI = Eyberg Child Behavior Inventory.

Table B2

Adjusted Linear Regression Model Predicting Change in ECBI Problem Scores in the Virtual PriCARE Trial

Predictor	B	SE	t	p	95% CI
(Intercept)	20.252	4.584	4.418	<0.001	[11.252, 29.251]
Baseline ECBI Problem Score	-1.507	0.131	-11.477	<0.001	[-1.765, -1.250]
Intervention	-10.018	2.116	-4.734	<0.001	[-14.172, -5.863]
Child sex (Male)	2.400	2.131	1.126	0.261	[-1.785, 6.585]
Caregiver sex (Male)	5.213	7.309	0.713	0.476	[-9.137, 19.564]
Child ethnicity (Non-Hispanic)	2.042	2.831	0.721	0.471	[-3.517, 7.601]
Child race (Other)	2.209	3.438	0.642	0.521	[-4.542, 8.960]
Child race (White)	-2.060	2.736	-0.753	0.452	[-7.432, 3.311]
Child age	-0.791	0.766	-1.033	0.302	[-2.295, 0.713]
Study Site (UNC)	0.965	2.302	0.419	0.675	[-3.554, 5.484]

Note. B = unstandardized regression coefficients representing the estimated mean change in ECBI Problem score associated with each predictor. Negative coefficients indicate greater reductions in child behavior problems. Model is adjusted for baseline ECBI Problem score, child age, child sex, caregiver sex, child race, child ethnicity, and study site (UNC vs. CHOP). ECBI = Eyberg Child Behavior Inventory.

Appendix C
Adjusted Linear Regression Models Comparing Delivery Mode Effects on Change in Eyberg Child Behavior Inventory (ECBI) Scores Across Virtual and In-Person PriCARE Trials

Table C1
Adjusted Linear Regression Model Predicting Change in ECBI Intensity Scores With Delivery Mode Comparison Across Virtual and In-Person PriCARE Trials

Predictor	B	SE	*t*	*p*	95% CI
Intercept	40.58	4.61	8.81	< .001	[31.54, 49.62]
Baseline ECBI Intensity Score	−0.37	0.02	−17.05	< .001	[−0.41, −0.32]
Intervention	−8.56	2.85	−3.01	.003	[−14.14, −2.97]
Delivery Mode (Virtual)	1.40	2.77	0.50	.614	[−4.04, 6.84]
Child Sex (Male)	1.26	1.64	0.77	.443	[−1.96, 4.48]
Caregiver Sex (Male)	5.20	4.83	1.08	.282	[−4.28, 14.69]
Child Ethnicity (Non-Hispanic)	2.43	2.24	1.08	.280	[−1.98, 6.83]
Child Race (Other)	−1.09	2.69	−0.40	.686	[−6.36, 4.19]
Child Race (White)	−2.80	2.03	−1.38	.169	[−6.78, 1.19]
Child Age	−0.13	0.60	−0.22	.823	[−1.30, 1.03]
Intervention × Virtual	−0.74	3.51	−0.21	**.833**	[−7.64, 6.15]

Note. B = unstandardized regression coefficients representing the estimated mean change in ECBI Intensity score associated with each predictor. Negative coefficients indicate greater reductions in ECBI Intensity scores. Model is adjusted for baseline ECBI Intensity score, intervention group, delivery mode (virtual vs in-person), child age, child sex, caregiver sex, child race, child ethnicity, study site and the intervention x delivery mode interaction. ECBI = Eyberg Child Behavior Inventory.

Table C2
Adjusted Linear Regression Model Predicting Change in ECBI Problem Scores With Delivery Mode Comparison Across Virtual and In-Person PriCARE Trials

Predictor	B	SE	*t*	*p*	95% CI
Intercept	3.21	1.01	3.19	.001	[1.24, 5.19]
Baseline ECBI Problem Score	−0.38	0.02	−15.96	< .001	[−0.43, −0.34]
Intervention	−2.42	0.69	−3.48	.001	[−3.78, −1.05]
Delivery Mode (Virtual)	−1.73	0.68	−2.56	.011	[−3.05, −0.40]
Child Sex (Male)	0.12	0.40	0.29	.772	[−0.67, 0.90]
Caregiver Sex (Male)	−0.18	1.18	−0.15	.880	[−2.49, 2.13]
Child Ethnicity (Non-Hispanic)	0.43	0.55	0.79	.428	[−0.64, 1.51]
Child Race (Other)	0.95	0.65	1.45	.148	[−0.34, 2.23]
Child Race (White)	−0.68	0.50	−1.37	.171	[−1.65, 0.29]
Child Age	0.36	0.15	2.49	.013	[0.08, 0.65]
Intervention × Virtual	0.28	0.86	0.33	.744	[−1.40, 1.96]

Note. B = unstandardized regression coefficients representing the estimated mean change in ECBI Problem score associated with each predictor. Negative coefficients indicate greater reductions in ECBI Problem scores. Model is adjusted for baseline ECBI Problem score, intervention group, delivery mode (virtual vs in-person), child age, child sex, caregiver sex, child race, child ethnicity, study site, and the intervention x delivery mode interaction. ECBI = Eyberg Child Behavior Inventory.

Appendix D

Consolidated Standards of Reporting Trials (CONSORT) Flow Diagram for Participant Enrollment, Allocation, Follow-up, and Analysis in the Virtual PriCARE Randomized Controlled Trial

Figure D1
CONSORT Flow Diagram for the Virtual PriCARE Randomized Controlled Trial

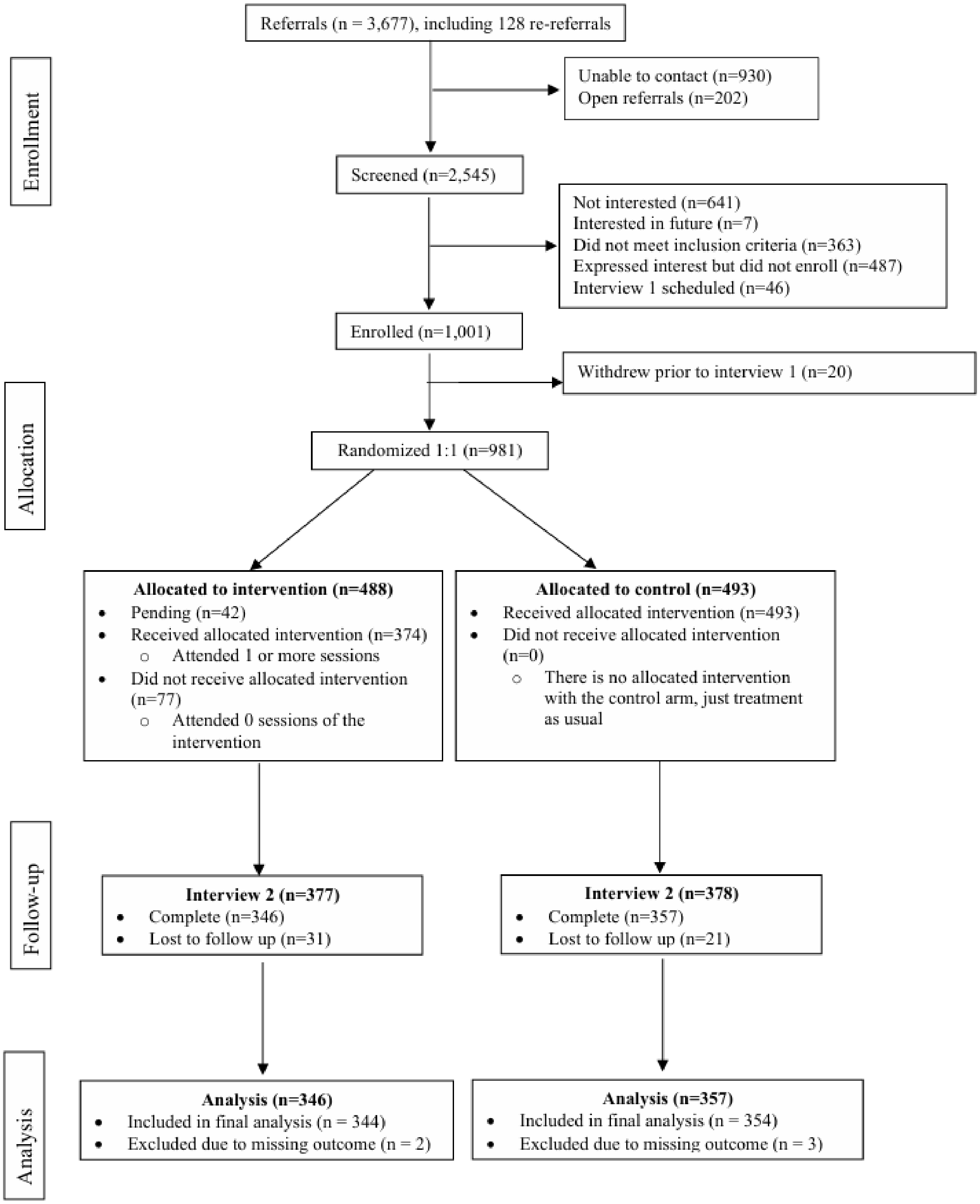

CONSORT flow diagram depicting participant referral, screening, enrollment, randomization, allocation to intervention or control, follow-up assessments, and inclusion in final analyses for the virtual PriCARE randomized controlled trial
